# Spatial Metabolomics and Its Application in Plant Research

**DOI:** 10.3390/ijms26073043

**Published:** 2025-03-26

**Authors:** Rong Li, Fang Wang, Jian Wang

**Affiliations:** 1 Academy of Agricultural and Forestry Sciences, Qinghai University, Xining 810016, China; rongli_ya@163.com (R.L.); qhwf324@163.com (F.W.); 2 Laboratory for Research and Utilization of Qinghai Tibet Plateau Germplasm Resources, Qinghai University, Xining 810016, China; 3 Key Laboratory of Qinghai-Tibet Plateau Biotechnology, Ministry of Education, Qinghai University, Xining 810016, China

**Keywords:** spatial metabolome, mass spectrometry imaging, spatial distribution

## Abstract

Spatial metabolomics, as a frontier technology, is capable of conducting the comprehensive characterization of metabolites within organisms in terms of qualitative, quantitative and positional dimensions, so as to facilitate the visual analysis of biological processes. This paper summarizes the birth and development of spatial metabolomics, explains its differences and advantages from traditional metabolomics and summarizes its application in plant research. In addition, the limitations of spatial metabolomics are summarized and discussed, along with the technological improvement and application innovation of spatial metabolomics, in order to provide reference for the development strategy of spatial metabolomics and its application in plant research.

## 1. Introduction

Plants contain extremely abundant metabolites with diverse chemical structures [1]. These endogenous metabolites not only play a role in maintaining growth development and generational reproduction but also play a role in resisting biotic and abiotic stresses and adapting to extreme environments [2]. In recent years, the biosynthesis, metabolic network, regulatory mechanism, genetic analysis and function study of metabolites in rice [3,4,5], wheat [6,7], maize [8,9] and other major crops have made rapid progress. However, the issue of the production and storage of metabolites in plants remains to be further explored. Therefore, the application of spatial metabolomics is of great significance for improving the depth of plant research and accelerating plant research progress.

Metabolomics was proposed by Nicholson [10] and Fiehn [11] at the end of the 20th century. It can identify and quantify metabolites such as alkaloids and flavonoids whose molecular weight is less than 1000 in living organisms [12]. German J B [13], a famous scholar, believes that “only metabolomics can truly reflect what has happened”. Compared with genomics, transcriptomics and proteomics, metabolomics can more directly reflect the current state of organisms [14], so as to more accurately explain phenotypic changes [15] and study the overall change rules [16]. Metabolomics is a science that studies the overall metabolites of an organism during different growth periods or before and after certain stimulation and interference conditions [17]. Nuclear magnetic resonance (NMR), liquid or gas chromatography (LC/GC), mass spectrometry (MS), chromatography–mass spectrometry and other techniques were used for comprehensive qualitative and quantitative analysis to reveal the accumulation patterns and change rules of metabolites [17]. Metabolomics was originally used to determine the dynamic changes in small-molecule metabolites in organisms caused by genetic or physiological and pathological changes [18,19]. At present, it has been widely used in the field of plants to determine the harvest site and time of plant-derived food or medicinal plants, to study the physiology of fruit ripening, to study the genetic mechanism of metabolic diversity in different parts and its correlation with complex traits of plants, and other aspects [20,21,22,23], which provide a foundation for the exploration of mature phenotypic mechanisms, the improvement in plant quality and the modification of breeding methods [24].

With the continuous deepening of life science research and the rapid progress of science and technology, more and more dimensions can be explored in the process of scientific research. Some novel technical means such as spatial transcriptomics, spatial proteomics and spatial metabolomics have gradually entered the field of vision. Spatial omics can provide multi-omics information such as transcriptome, proteome and metabolomics on the premise of retaining the original spatial position of tissues, organs and cells of organisms, and reveal the microenvironment interaction and spatial heterogeneity between cells from a molecular perspective, which provides an innovative research tool for biomedicine and other research fields [25]. Spatial transcriptomics and spatial proteomics are used to analyze the molecular mechanism at the molecular level, but spatial metabolomics can effectively link the biological tissue and cell level with the molecular level, so it is especially crucial. As an emerging analytical technique, spatial metabolomics takes in situ metabolites of organisms as the research object, and integrates mass spectrometry imaging (MSI) and metabolomics technology. The different distribution of various metabolites in biological tissues and cells was accurately identified and located through MSI technology, and in-depth metabolomics analysis was performed on the target micro-region tissue by metabolomics technology to obtain metabolite type and content information [26,27,28]. The working principle of MSI technology is to desorb and ionize the appropriate ion source on the surface of the sample to obtain the ionic strength of various molecules in each pixel of the sample and then reconstruct the spatial in situ distribution information of various molecules in the sample with the help of specific imaging software [29]. Spatial metabolomics has been widely used in the fields of medicine and animals, and its application in the field of plants is still at an initial stage nowadays. In plant research, the concept of space is of great significance. The synthesis and accumulation of metabolites in plants generally have accurate spatial distribution, and physiological functions are usually closely related to their spatial distribution in tissues and organs or even single cells [1]. Therefore, accurate positioning of metabolite distribution is crucial to elucidate the synthesis, accumulation and regulation mechanism of metabolites in plants. In this context, spatial metabolomics has potential application value and broad application prospects in the field of plant research.

This paper primarily summarized the birth and development of spatial metabolomics and explained its differences and advantages from traditional metabolomics. Secondly, the application of spatial metabolomics in plant research was reviewed and summarized. Finally, the limitations of spatial metabolomics were summarized and discussed, and its technical improvement and application innovation in plant research were prospected. The purpose of this paper is to introduce the basic concepts and development process of spatial metabolomics, and to illustrate its importance in the field of plant research with its unique advantages, so as to raise the awareness of researchers to carry out plant molecular biology research with new technologies, and lay a foundation for plant breeding and improvement.

## 2. Research Progress of Spatial Metabolomics

### 2.1. The Birth of Spatial Metabolomics

**Figure 1:**
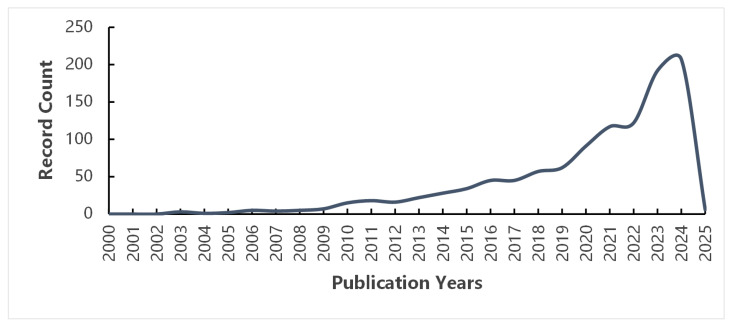
The literature statistics related to spatial metabolomics since 2000.

Tissues and organs in organisms are composed of polytype cells and of heterogeneity and complexity. The spatial distribution of metabolites is closely related to their physiological and pathological functions, so it is necessary to link the dynamic changes in metabolites with the spatial location of changes [30]. Traditional metabolomics cannot provide the dimension of space. Nowadays, the rapid development of MSI technology has contributed to the birth of spatial metabolomics. The emergence of this method makes up for the limitations of the loss of spatial distribution information of traditional metabolomics, effectively solves the biological problems of micro-area heterogeneity and then improves the comprehensiveness of metabolite research [31]. In this paper, it is observed that the number of publications on spatial metabolomics has exhibited a consistent upward trend since 2000 through Web of Science (Figure 1). Since 2021, the annual number of publications has exceeded 100, indicating a growing interest in the application of spatial metabolomics, and it is one of the hot research fields at present and in the next few years.

### 2.2. Spatial Metabolomics and Mass Spectrometry Imaging Technology

Spatial metabolomics is a research method based on the development of MSI technology, whose birth and further development are inseparable from MSI technology. MSI technology is a new type of molecular imaging technology that emerged along with the development of mass spectrometry, which was first used to study the distribution of proteins in biological tissues in 1997 [32]. The chemical information and spatial information of various samples can be obtained simultaneously by mass spectrometry and then used as chemical images for subsequent processing and visualization [33,34,35]. Since the birth of the first mass spectrometer in 1912, MSI technology has experienced more than one hundred years of development. Especially in recent decades, with the application of new mass analyzer and ionization technology, MSI technology has been rapidly developed, whose mass resolution, ion acquisition speed and accuracy have been greatly improved [36]. MSI technology can realize the qualitative, quantitative and localization analysis of thousands of metabolites in biological tissues. Combined with bioinformatics analysis, it has developed into spatial metabolomics, which can find different metabolites in situ from biological tissues [34,37] to further identify and verify their biological functions [28].

The conventional workflow of MSI technology can be divided into three steps: sample preparation, mass spectrometry data acquisition and visual analysis [38,39]. (1) Sample preparation: The sample is collected, embedded and sliced to become a thin and stable sample slice [40]; (2) mass spectrometry data acquisition: The sample area to be imaged is divided into a two-dimensional lattice composed of several points. A focused ionization source is used to bombard the sample slice point by point, so that the molecules in the slice are desorbed and ionized. Then, the charged ions enter the mass spectrometer, so as to obtain the mass-to-charge ratio, coordinates and signal intensity data of each point, which will be transformed into the pixels of the imaging map [41]; (3) visual analysis: The information of each site on the sample surface can be obtained by mass spectrometer analysis, and then the two-dimensional distribution map of the corresponding molecules or ions on the sample surface can be drawn by imaging analysis software [42].

According to the different ionization modes, MSI technology mainly includes matrix-assisted laser desorption ionization mass spectrometry imaging (MALDI-MSI) technology, secondary ion mass spectrometry imaging (SIMS-MSI) technology, desorption electrospray ionization mass spectrometry imaging (DESI-MSI) technology and other technologies. MALDI-MSI technology is an analysis method based on soft ionization MALDI, which is currently the most widely used MSI technology. Its limitations are mainly due to the time-consuming pre-processing and the high cost of laser maintenance [43]. SIMS-MSI technology is an analysis method based on nanomaterials, which is the earliest MSI technology used to analyze surface materials [44]. DESI-MSI technology is an analysis method that can be carried out at atmospheric pressure [45], which is an MSI technology commonly used today, and it is also another major breakthrough in the development history of mass spectrometry ionization technology, opening a new era of atmospheric mass spectrometry. Benefiting from no laser and a simple ion source structure, DESI-MSI has low maintenance costs [46]. Table 1 compares MALDI-MSI, SIMS-MSI and DESI-MSI from the aspects of detectable substance type, mass range and spatial resolution [29].

### 2.3. Advantages of Spatial Metabolomics

Spatial metabolomics can be used for qualitative, quantitative and locational analysis of metabolites. It is a research method that directly obtains the types, contents and spatial distribution information of metabolites from organisms to describe the metabolic network diagram [31]. Spatial metabolomics enables researchers to simultaneously obtain biomolecular chemical information and spatial information, to better understand the heterogeneity and complexity of organisms, and then further explore the mechanism, which has become a powerful tool to understand the spatial organization and molecular interaction of biological systems [47].

The advantages of spatial metabolomics compared to traditional metabolomics are mainly reflected in three aspects: high accuracy, high richness and strong comprehensiveness (Table 2).

## 3. Application of Spatial Metabolomics in Plant Research

The emergence of spatial metabolomics makes up for the shortcomings of traditional metabolomics and realizes the accurate positioning of metabolites in tissues with high spatial resolution. Spatial metabolomics has been widely used in the study of the spatial component distribution of plants, mainly involving medicinal plants, food crops and economic crops, but its application purposes in different plant types are disparate (Table 3).

### 3.1. Application of Spatial Metabolomics in Medicinal Plants

Traditional Chinese medicine has the characteristics of being multi-component, multi-target and multi-pathway [52]. Its complex material basis and diverse mechanism of action bring difficulties to the research of traditional Chinese medicine [53]. Traditional Chinese medicine analysis methods first powder the medicinal materials and then use the medicinal powder as raw material to extract natural components, which often needs to be treated by various methods such as decoction and reflux [54]. The classical pretreatment process is time-consuming, the sample preparation is cumbersome and a large amount of organic solvents are consumed, which can easily cause the decomposition and loss of Chinese medicine components. Since the research object is the medicinal powder, the analysis results will inevitably lose the spatial distribution information of the compound in the plant tissue [53]. Spatial metabolomics technology can greatly reduce the complexity of pretreatment while retaining the spatial distribution information of compounds in plant tissues, making the analysis results more accurate and comprehensive. At present, the application of spatial metabolomics technology in medicinal plants is mainly reflected in the visual analysis of natural components and the quality control of medicinal materials.

#### 3.1.1. Visual Analysis of Natural Ingredients

The material basis of traditional Chinese medicine is complex and exquisite. Single medicinal materials contain a variety of natural compounds with different structures and different physical and chemical properties, which make it difficult to analyze the components of traditional Chinese medicine [53]. In recent years, spatial metabolomics technology has been used for the visual analysis of natural components of traditional Chinese medicine (Table 4). Paris polyphylla Smith var. yunnanensis has high medicinal value. In the study of its components, liquid chromatography/liquid chromatography–mass spectrometry (LC/LC-MS) is usually used to qualitatively or quantitatively analyze the extract of medicinal materials [55]. However, this technology has certain defects. Complex sample pretreatment will lose the content of metabolites in the extract, resulting in difficulty in detection, and the spatial distribution of metabolites cannot be displayed. The application of MALDI-MSI technology for the first time characterized the spatial distribution of metabolites in the rhizome of Paris polyphylla Smith var. yunnanensis, and provided a reference for the identification of *Paris polyphylla Smith* var. *yunnanensis*, the extraction and separation of metabolites and the exploration of the ‘time-space’ law of metabolites [56]. Spatial metabolomics technology can obtain the spatial distribution information while identifying the natural components of traditional Chinese medicine, so as to analyze the natural components of traditional Chinese medicine in situ in a simple, rapid and direct manner, thus providing a new and effective method for revealing the material basis of traditional Chinese medicine.

#### 3.1.2. Quality Control of Medicinal Materials

The quality control of traditional Chinese medicine is an important guarantee to ensure the clinical efficacy. The composition, content and distribution of medicinal parts can directly reflect the quality of traditional Chinese medicine. Compared with traditional analysis methods, spatial metabolomics is used to characterize the components in situ, so as to evaluate the varieties, origin, growth years, picking period and processing of traditional Chinese medicine. It has broad application prospects in the quality control of traditional Chinese medicine [18]. Spatial metabolomics links morphological or quality information with chemical composition characteristics, clearly and intuitively reveals the accumulation sites of traditional Chinese medicine components in different tissues and provides a new reference for identifying key index components, exploring the synthesis mechanism of pharmacodynamic components, improving the extraction process of medicinal materials and revealing the molecular mechanism of toxicity. This technology plays a role in the development and utilization of traditional Chinese medicine, so as to improve the medicinal value of traditional Chinese medicine and improve the quality control system of medicinal materials.

In the empirical identification, the quality of *Paeoniae Radix Alba* and *Paeoniae Radix Rubra* is evaluated by root thickness, and paeoniflorin serves as a common quality indicator of them, but the correlation between the content of bioactive compounds and the root size is still unclear. The distribution of bioactive compounds in different tissues on the cross-section of the root was analyzed by DESI-MSI technology. The results indicated that paeoniflorin and albiflorin, with the largest differences in the xylem and the bark of the roots, had the highest content in the two tissues. The root diameter was positively correlated with the paeoniflorin content and negatively correlated with the albiflorin content. As isomers with different efficacies, paeoniflorin or albiflorin can be chosen as the quality marker corresponding to specific clinical applications to launch the quality classification evaluation of multi-functional Chinese medicines [78]. As a precious traditional Chinese medicine, *Cordyceps sinensis* is in short supply due to the decline in natural yield and the increase in human demand. Cultured Cordyceps sinensis (CCS) has gradually entered the market. However, traditional analytical techniques can only evaluate specific components extracted by specific solvents and cannot guarantee its quality, resulting in its price being far from that of natural Cordyceps sinensis (NCS). To solve this problem, the in situ chemical composition analysis of NCS and CCS was carried out by TOF-SIMS technology. Data showed that CCS has comparable proportions of amino acids, nucleosides, monosaccharides, sphingolipids, sterols and other principles to NCS except for fatty acids, glycerides and glycerophospholipids, which revealed the high chemical similarity between CCS and NCS [79]. It is expected to alleviate the dilemma of NCS, including endangered species, the impact of soaring prices and ecological problems caused by excessive harvesting. In addition, the researchers used spatial metabolomics to characterize the ‘space-content’ of traditional Chinese medicine. The in situ characterization of *Panax ginseng* with different growth years [63,81], *Forsythia suspensa* with different harvest periods [82] and *Salvia miltiorrhiza* Bge with continuous cropping [83] showed that different growth years, harvest periods and planting methods will lead to changes in the composition of traditional Chinese medicine, which provides an important basis for improving the quality of traditional Chinese medicine. The in situ characterization of the steaming process of *Gastrodiae Rhizoma* [84] and *Aconitum carmichaelii* Debeaux [85] showed that the processing technology would affect the spatial distribution of traditional Chinese medicine components, which provided theoretical support for the standardized processing of and quality improvement in traditional Chinese medicine. The in situ characterization of rat kidney slices after administration of aristolochic acid I shows that different organs have different sensitivity to the toxicity of traditional Chinese medicine [86]. It provides a new strategy for determining the toxic target organs, revealing the toxic material basis and molecular mechanism, which is of great significance for the toxicity-reducing and action-enhancing ability of traditional Chinese medicine, clinical rational application and the development of low-toxic new drugs.

### 3.2. Application of Spatial Metabolomics in Food Crops and Economic Crops

#### 3.2.1. Assisting Specificity Identification

Spatial metabolomics can obtain the spatial chemical information of metabolites in specific regions of different tissues, so as to observe the metabolites in tissues more intuitively, provide a new means for the identification of tissue specificity and material specificity and ultimately serve the extraction and separation of metabolites and the exploration of their regulatory rules, the strict distinction of specific materials and their innovative use in breeding. The spatial distribution of major lipids in *Brassica napus* seeds was studied by MALDI-MSI technology. The results showed that the lipid composition was significantly different among different tissues, suggesting that the regulation of seed lipid metabolism was tissue-specific. Further analysis of the differential regulation will help to improve the yield and quality of Brassica napus [87]. Studies on the complex spatial distribution patterns of lipid metabolites in *Brassica napus* showed that triacylglycerol and phosphatidylcholine were unevenly distributed in embryos, revealing the tissue specificity of lipid metabolism in high- and low-oil Brassica napus seeds [88].

#### 3.2.2. Analyzing Anabolic Pathways of Metabolites

More than 200,000 metabolites exist in plants, including primary metabolites necessary for maintaining growth, development and life activities, as well as secondary metabolites related to disease resistance and stress resistance [89]. Accurate localization and quantification of metabolites are of great significance for elucidating the regulatory mechanism of metabolites in plants. At present, plant imaging techniques, such as optical microscopy and electron microscopy, have been used for morphological characterization and chemical localization of marker components in plant tissues. Currently, plant imaging techniques, such as light and electron microscopy, are used for morphological characterization and chemical localization of labeled components in plant tissues. However, there are significant limitations using these techniques as they require the labeling of specific chemicals, limiting de novo investigation of unlabeled and unknown chemical compounds [70]. Therefore, it is difficult to determine the spatial distribution of a single unlabeled or unknown metabolite by microscopy, and the clear localization of the synthesis, transport and accumulation of many metabolites in tissues has not yet been achieved. Spatial metabolomics can provide a direct link between tissue structure and individual metabolites, which provides a new idea for further exploring the biosynthesis, transportation and accumulation of plant metabolites. Spatial metabolomics is easy to operate while ensuring the in situ spatiality and accuracy of the analyte. It has broad application prospects in the study of the spatial distribution of plant metabolites and has great potential in the study of the synthetic metabolic pathways of plant metabolites. It is of great significance for the study of the spatial distribution and synthetic metabolic pathways of key components.

Mass spectrometry imaging was used to interrogate the spatio-chemical localization of metabolites with matrix-assisted laser desorption/ionization and laser desorption/ionization. Fourier-transform ion cyclotron resonance MS across the *Ginkgo biloba* leaf combined with gene expression analysis suggested that the site of ginkgolide biosynthesis is localized in the root, and the products are then translocated and accumulated mainly in the leaf tissues, which further deepened the study of *Ginkgo biloba* leaf metabolites [70]. The similarities and differences in phytochemistry and pharmacological effects of the only genus in the family *Paeoniaceae*, *Paeonia suffruticosa* (PS) and *Paeonia lactiflora* (PL) have been extensively investigated. MALDI-MSI was used to characterize the spatial distribution of the roots of PS and PL. It was found for the first time that there were significant differences in the spatial distribution of 13 major intermediates involved in the gallotannin biosynthetic pathway in the roots of the two medicinal materials. In this work, the biosynthetic pathway of gallotannins in PS and PL was visualized with MALDI MSI for the first time, and provides important information for the study of the biosynthesis, transportation and accumulation of Paeoniaceae metabolites. The changes in its spatial metabolome are of great significance in the fields of plant taxonomy, Paeoniaceae evolution and quality control of medicinal plant materials [59]. In addition, the MALDI-MSI method was used to analyze the specific changes in tomato root exudates induced by different microbial communities. The results showed that the metabolites were located in specific and different regions, rather than evenly distributed in the root system [90]. The visual characterization of *Glycyrrhizae Radix et Rhizoma* decoction pieces by DESI-MSI technology is conducive to understanding and mastering the distribution law of specific components and in vivo biosynthesis [91].

#### 3.2.3. Exploring the Regulation of Growth and Development

Spatial metabolomics has realized endogenous molecular imaging in botany research, filled up the spatial distribution information of endogenous molecules that cannot be presented by traditional techniques, provided great potential for better understanding of various physiological processes of plants and provided a powerful research method for elucidating the dynamic changes in metabolites in different growth and development processes of plants [92]. Traditional metabolomics is an effective method to understand the overall metabolic changes in plant tissues. Gas chromatography–mass spectrometry (GC-MS) and liquid chromatography–mass spectrometry (LC-MS) are used to detect various metabolites that appear at the same average level throughout the tissue. However, it does not clarify more local physiological phenomena, such as metabolic responses to abiotic or biotic stresses at the micro-regional level of the tissue. Spatial metabolomics can more accurately understand the physiological changes and responses of tissue microregions, which is of great significance in exploring the regulation of plant growth and development.

Tomato (*Solanum lycopersicum* L.) is an edible horticultural crop that is highly consumed worldwide and is used as a model plant for mature research [93]. In order to study the temporal and spatial metabolic changes in the process of tomato physiological changes at the overall tissue level, MALDI-MSI technology was used to study the different mature phenotypes of mature green and mature red tomato fruits, which increased the understanding of tomato fruit physiological changes [94]. MALDI-MSI technology has been widely used in the development process. The application of MALDI-MSI in the development of wolfberry fruit showed that with the development of fruit, the signal intensity of citric acid decreased, while the signal intensity of choline, betaine, hexose and sucrose increased. During the development process, a variety of phenolic acids and flavonoids were accumulated in the exocarp, and it was speculated that it had a protective effect on abiotic and biotic stress [95]. The application in the development of rape seeds showed that spermidine conjugates played an important role in the development of rape hypocotyls, which then differentiated into plant roots and stems [96]. The application in maize inbred lines B73 and Mo17 and their reciprocal hybrids revealed that there were differences in the abundance and distribution of amino acids in the roots of the two maize varieties, and the hybrids showed the characteristics of parental inheritance [97]. In addition, MALDI-TOF technology was used to analyze the frontal surface of the blue petals of *Viola cornuta*, which contained one anthocyanin pigment (violanin) and a number of flavonols acting as potential copigments to further elucidate the molecular mechanism of blue flower coloration [98]. The distribution differences in citric acid, soluble sugar and anthocyanin in strawberry at different maturity stages were observed by MALDI-TOF-MS. The results showed that citric acid and sugar were evenly distributed in each maturity stage of the whole fruit, while anthocyanin was mainly distributed in the periphery of the fruit, and the abundance increased in red strawberry, which indicated that it was related to color attributes [99].

#### 3.2.4. Revealing the Interaction Mechanism Between Crop and Environment

Spatial metabolomics has been used to elucidate the mechanism of disease induction and insect infestation and explore the best processing methods, and may help to decipher the role of signal molecules in plant abiotic and biotic stress responses. The application of spatial metabolomics in the mechanism of disease and insect infestation and the exploration of the best processing methods explains the previous judgments and choices made by experience. It can be seen that it plays an important role in revealing the interaction mechanism between plants and the environment, and is expected to solve the difficulties caused by the interaction of multiple factors in the interaction research.

MALDI-MSI technology is currently the main application in revealing the mechanism of plant–environment interaction. A large number of secondary metabolites located and identified in the rhizome tissues of *Glycyrrhiza glabra* verified the function of flavonoids in plant defense [57]. The spatial difference distribution of secondary metabolites, endogenous polypeptides and proteins in tomato roots infected and uninfected by root-knot nematodes showed that the expression of some polypeptides and proteins was down-regulated after root-knot nematodes infection, and the types and abundance of lipid metabolites were also significantly decreased [100]. The metabolic mechanism of host–virus interaction between *Emiliania huxleyi* and its specific viruses has been revealed, and the entire viral infection process has been dynamically monitored using chlorophyll and seven lipid molecules as biomarkers [101]. The DESI-MSI technique was used to study the effects of mechanical damage on the levels of plant hormones and metabolites in *Arabidopsis thaliana*. It was found that compared with the control, the damaged leaves showed specific accumulation of jasmonic acid, salicylic acid, abscisic acid and auxin; especially jasmonic acid was strongly induced by damage [102]. MALDI-MSI was applied to *Arabidopsis thaliana* leaves and the major glucosinolates were found to be more abundant in tissues of the midvein and the periphery of the leaf than the inner lamina, which appears to explain the feeding preference of *Helicoverpa armigera* larvae for specific portions of the leaf [103]. The differential distribution of glucosinolate metabolites may play an important role in the defense mechanism of leaves, and to avoid the issue that herbivores or insects often start eating from the edge of leaves. This study is of great significance for anti-herbivorous defense. Combined targeted metabolite profiling with matrix-assisted laser desorption/ionization–mass spectrometry imaging was employed to investigate the accumulation of dhurrin, its recycling products and key general metabolites in four different sorghum lines during 72 h of grain imbibition, germination and early seedling development, as well as the spatial distribution of these metabolites in two of the lines. Matrix-assisted laser desorption/ionization–mass spectrometry imaging demonstrated that dhurrin primarily accumulated in the germinating embryo, confirming its function in protecting the emerging tissue against herbivory [104].

#### 3.2.5. Verifying Gene Function

Spatial metabolomics has been shown to be able to combine with reverse genetics to elucidate gene function, and it may be applied to gene function verification in the future. Overexpression, silencing and other transgenic materials usually lead to the change of only one gene. The polygene hypothesis indicates that a gene is difficult to cause changes in plant phenotype, yield or resistance. Therefore, determining a transgenic material and verifying its function has become a difficult point in transgenic breeding. Spatial metabolomics can be used to visualize the spatial distribution differences between transgenic materials and wild-type materials in situ, and local small changes are more likely to be detected, so as to assist the positive identification of transgenic materials and the verification of gene function. The MALDI-MSI method was used to visualize the synthesis pathway of steroidal glycoalkaloids (SGAs) in tomato fruits. By visualizing and quantifying SGA spatial differentiation in wild-type and GAME25i tomato fruits at the mature red ripe stage, it was found that upon silencing of the GAME25 gene, the levels of α-tomatine and its downstream saturated SGA derivatives were all significantly reduced compared with wild-type fruit. By contrast, the levels of dehydrotomatine and its downstream unsaturated SGA derivatives were all increased in mature red ripe GAME25i fruit. In particular, MALDI images showed that saturated SGAs and their respective unsaturated forms exhibited very similar distribution patterns in wild-type and GAME25i fruit. During conversion of α-tomatine to esculeosides, and dehydrotomatine to dehydroesculeosides, saturated and unsaturated SGA intermediates mostly accumulated in the epidermis layer of both wild-type and GAME25i fruit. Semiquantitative and distribution information together suggested that silencing of GAME25 reroutes the entire saturated SGA repertoire towards the unsaturated SGA branch, while the spatial distribution of saturated SGAs and their respective unsaturated forms remain the same in wild-type and GAME25i fruit [105].

### 3.3. Advantages and Disadvantages of the Three Main MSI Technologies in Spatial Metabolomics

The application of spatial metabolomics in different plants or tissues shows that the three main MSI techniques have their own advantages and disadvantages. Among them, MALDI-MSI is an effective molecular imaging technology, which has the advantages of low sample requirements, without extraction, high resolution, without pre-labeling, and in situ analysis [56]. However, due to its limited sensitivity, it is difficult to perform global imaging of all small-molecule metabolites. Therefore, it is possible to pretreat slices to remove interfering substances such as salt and fat-soluble components as much as possible, minimizing ion suppression, such as short-term immersion and rinsing of slices [43]. With the advantages of controllable structural properties, diverse types and excellent performance of nanomaterials, SIMS-MSI has achieved high-resolution, high-sensitivity, selective and universal mass spectrometry detection imaging. However, it needs to be carried out in a closed vacuum environment, and the selection of the imaging area is stiff, which cannot achieve real-time analysis and dynamic analysis of the reaction process [44]. The atmospheric mass spectrometry technology based on this problem has also shown a strong momentum of development in the field of imaging in recent years [56]. DESI-MSI technology has the advantages of being without vacuum conditions, without matrix coverage and having low requirements for mass spectrometers. However, the ionization efficiency of non-polar compounds is very low, and the signal suppression of high-salt tissue sections is very obvious [45].

At present, MSI technology still has some limitations that cannot be ignored [29]. For example, the lower molecular ionization level limits the realization of higher resolution; the higher scientific research cost, longer test cycle and complex data analysis hinder the application of more fields. More open-source, practical and powerful bioinformatics tools and data processing methods are urgently needed to help MSI technology achieve more extensive and comprehensive application.

## 4. Summary and Outlook

Spatial metabolomics is a new method of omics research based on mass spectrometry imaging technology in recent years. It makes up for the shortcomings of traditional metabolomics in situ visualization analysis and has achieved many research results in disease diagnosis and treatment, traditional Chinese medicine analysis, pharmacological metabolism and other aspects. It has become a research hotspot in medicine and other fields [106]. The introduction of spatial metabolomics technology into plant research can provide a new perspective for specific identification, synthetic metabolic pathways, growth and development rules, interaction mechanisms and gene function research. While spatial metabolomics has made significant progress, several challenges remain that must be addressed to fully unlock its potential. In the future, in-depth research can be considered from the following aspects: (1) Spatial metabolomics has not yet formed unified and standardized operating guidelines in sample preparation, data collection and processing and other key operations, resulting in poor reproducibility of experimental results. Especially in sample preparation, any subtle differences such as the thickness of the tissue section, the staining process and even the storage conditions of the tissue sample may lead to deviations and affect the final experimental results. Therefore, it is necessary to establish a standardized spatial metabolomics operation process and quantitative analysis methods; (2) spatial metabolomics uses a mass spectrometer to capture the accurate mass spectrometry information of metabolites in tissue sections. The sensitivity and resolution of the instrument are crucial for subsequent analysis, and improving the detection resolution will greatly prolong the imaging time. Therefore, a new type of high-sensitivity mass spectrometry imaging device that can take into account both a high resolution and imaging speed is urgently needed; (3) the comprehensive application of spatial transcriptomics, spatial proteomics and spatial metabolomics can construct a multi-dimensional bioinformatics map, providing a comprehensive perspective from gene expression to functional proteins, and then to cell metabolism. This comprehensive analysis can not only reflect the interaction between different biological levels but also provide a more in-depth understanding of biological processes and disease mechanisms; (4) the existing mass spectrometry imaging technology has poor universality. For samples with high moisture and starch content, it is easy to cause the loss of local composition and location information. This problem limits its application range. It is urgent to further improve it to be widely applied to different types of samples and solve the problem of research bottlenecks in various fields; (5) the existence of some special structures in plant cells, such as a cuticle and wax layer, hinders the detection and analysis of endogenous metabolic molecules in plant leaves, which makes it difficult for mass spectrometry imaging. In addition, it is impossible to achieve rapid and sensitive in situ analysis of single cells [107]. Therefore, it is urgent to further improve the existing technology to solve these problems.

Spatial metabolomics provides a new way for the study of plant growth and development and the regulation of spatial metabolic networks, and provides key support for solving problems in agricultural production, plant energy development and other fields. In the future, the application of plant spatial metabolomics technology can not only play an important role in basic research but also provide an important theoretical basis and practical guidance for plant breeding improvement and agricultural production. In summary, a giant development space still exists in the application of spatial metabolomics technology in the field of plants. It will inevitably further promote the modernization development of plants and even the entire biological field after continuous improvement and innovation.

## Figures and Tables

**Table 1:** Comparison between different MSI techniques.

Technology	MALDI MSI	SIMS MSI	DESI MSI
Ionization type	Soft	Hard	Soft
Need matrix or not	Need	Not	Not
The type of substance that can be detected	Small-molecule metabolites, drugs, biological macromolecules such as peptides, proteins, nucleic acids, polysaccharides	Small-molecule metabolites, drugs, lipids, elements	Small-molecule metabolites, drugs, lipids, peptides
Mass range	300–100,000 Da	<2000 Da	100–2000 Da
Spatial resolution	5–100 μm	0.1–1 μm	40–200 μm
Depth of the scanning	0.1–20 μm	0.5–10 μm	1–50 μm

**Table 2:** Comparison between spatial metabolomics and traditional metabolomics.

Advantages of Spatial Metabolomics	Limitations of Traditional Metabolomics	Improvement of Spatial Metabolomics
The sampling process of spatial metabolomics is relatively simple so that the test results have high accuracy.	Traditional metabolomics usually requires sample pretreatment such as tissue homogenate, metabolite extraction and purification and enrichment operations such as solid-phase extraction before detection, which will lead to differences between the sample to be tested and the original state [31].	Spatial metabolomics does not require special treatment before sample detection. After the sample is simply cleaned or wiped clean, it can be embedded with reagents such as frozen section embedding agent (OCT), carboxymethyl cellulose (CMC) and gelatin [43], which maintain the original state of the metabolite to a greater extent to improve the accuracy of the test results.
Spatial metabolomics has a wider range of applications and can detect more abundant substances.	The detection sensitivity of traditional metabolomics is relatively low, and it is difficult to detect metabolites with large differences in high throughput at the same time [48].	Spatial metabolomics can detect thousands of metabolites at the same time because of its high sensitivity, high coverage and high resolution, so it is easier to detect differential metabolites in some metabolic pathways [49].
The spatial distribution information of spatial metabolomics is clear, and the analysis dimension is comprehensive.	Traditional metabolomics only detects the quality and quantity of metabolites, and lacks the spatial distribution information of metabolites in the original tissues and organs [50]. However, the spatial distribution information is very important for the study of the overall effects of physiological functions.	Spatial metabolomics is embedded in the original state of the sample, which can describe the specific location of changes in the micro-area directly related to the research target [51], and raise the metabolomics information from the two-dimensional level to the three-dimensional level, providing visual data information for plant research.

**Table 3:** Application of spatial metabolomics in different plant types.

Plant Types	Application Purposes
Medicinal plants	Visual Analysis of Natural Ingredients
	Quality Control of Medicinal Materials
Food Crops and Economic Crops	Assisting Specificity Identification
	Analysising Anabolic Pathways of Metabolites
	Exploring the Regulation of Growth and Development
	Revealing the Interaction Mechanism between Crop and Environment
	Verifying Gene Function

**Table 4:** Application of spatial metabolomics technology in visual analysis of natural components of traditional Chinese medicine.

Medicinal Plants	Tissue Sites	Natural Components	MSI Technology	Reference
*Paris polyphylla Smith* var. *yunnanensis*	Rhizome	Steroid saponin, amino acids, organic acids, sterols, ecdysterone, nucleosides, esters	MALDI	[56]
*Glycyrrhiza glabra*	Rhizome	Free flavonoids, flavonoid glycosides and saponins	MALDI	[57]
*Paeonia lactiflora*	Root	Gallotannins and monoterpene glucosides	MALDI	[58]
*Paeonia lactiflora*	Root	Monoterpene, paeonol glycosides, tannins, flavonoids, saccharides and lipids	MALDI	[59]
*Tripterygium*	Root	Triterpenoids and sesquiterpene alkaloids	MALDI	[60]
*Curcuma longa*	Root	Curcumin	MALDI	[61]
*Panax*	Root	Saponins	MALDI	[62]
*Panax ginseng*	Root	Ginsenosides	MALDI	[63]
*Salvia miltiorrhiza*	Root, stem	Amino acids, phenolic acids, fatty acids, oligosaccharides, cholines, polyamines, tanshinones and phospholipids	MALDI	[64]
*Salvia miltiorrhiza*	Root, stem, leaf	Salvianolic acids, tanshinones	MALDI	[65]
*Panax notoginseng*	Root	Notoginsenosides, ginsenosides, amino acids, dencichine, gluconic acid and low-molecular-weight organic acids	MALDI	[66]
*Angelica pubescens*	Root, velamen	Coumarins	MALDI	[67]
*Aquilaria sinensis*	Stem	(2-phenylethyl) chromones and their analogs	MALDI	[68]
*Morus alba*	Leaf	Protocatechuic acid, chlorogenic acid, monosaccharide, disaccharide, astragalin, rutin, isoquercetin, cyanidin-3-O-glucoside, quercetin-3-O-6″-O-acetyl-β-D-glucopyranoside and kaempferol-3-O-rutinoside	MALDI	[69]
*Ginkgo biloba*	Leaf	Flavonoids, ginkgolic acids, cardanols, saccharides, phospholipids, chlorophylls, ginkgolides	MALDI	[70]
*Ligustri Lucidi Fructus*	Fruit	10-hydroxyoleoside dimethylester, 8-demethyl-7-ketoliganin, elenolic acid, salidroside, neonuezhenide/isomer, verbascoside/isomer, luteoline, nuzhenal A	MALDI	[71]
*Dendrobium nobile*	Stem	Alkaloids, sesquiterpenoids	MALDI	[72]
*Hypericum*	Flower, leaf	Hypericin	MALDI	[73]
*Isatidis Radix*	Root	3-formylindole, epiprogoitrin/progoitrin, isatithioetherin C/isatithioetherin E, coniferin, syringing, clemastanin B, adenosine, adenine, uridine, arginine, malic acid, maleic acid/fumaric acid, citric acid, emodin-8-O-β-D-glucoside and isovitexin	MALDI/DESI	[74]
*Radix Scutellariae*	Root	Baicalein and wogonin	PALDI	[75]
*Mentha piperita*	Leaf	Flavonoids	DESI	[76]
*Datura leichhardtii*	Leaf	Alkaloids atropine, scopolamine	DESI	[77]
*Paeonia lactiflora*	Root	Paeonol glycosides, albiflorin	DESI	[78]
*Cordyceps sinensis*	Caterpillars	Fatty acids, glycerides, Glycerophospholipids, amino acids, nucleosides, monosaccharides, sphingolipids, sterols	SIMS	[79]
*Coptis chinensis*	Rhizome	Berberine, epiberberine, coptisine, palmatine, columbamine, jatrorrhizine, tetrahydricheilanthifolinium, oxyberberine	SIMS	[80]
